# Radiological Evaluation Criteria for Chronic Subdural Hematomas: Review of the Literature

**DOI:** 10.1007/s00062-022-01138-1

**Published:** 2022-02-14

**Authors:** Matthias Bechstein, Rosalie McDonough, Jens Fiehler, Umberto Zanolini, Hamid Rai, Adnan Siddiqui, Eimad Shotar, Aymeric Rouchaud, Mayank Goyal, Susanne Gellissen

**Affiliations:** 1 Department of Diagnostic and Interventional Neuroradiology, University Medical Center Hamburg-Eppendorf, Martinistraße 52, 20246 Hamburg, Germany; 2 Department of Neurosurgery, University at Buffalo, Buffalo, NY USA; 3 Neuroradiology Department, Pitié-Salpêtrière Hospital, Paris, France; 4 Neuroradiology Department, Dupuytren, University Hospital of Limoges, Limoges Cedex, France; 5 Department of Radiology, University of Calgary Cumming School of Medicine, Calgary, AB Canada

**Keywords:** Subdural hematoma, Intracranial hemorrhage, Embolization, Middle meningeal artery, Midline shift

## Abstract

**Background:**

The methodology of measuring chronic subdural hematoma (cSDH) extent and its effect on intracranial structures is relevant for patient classification and outcome measurements and affects the external validity of cSDH studies. With embolization of the middle meningeal artery (MMA) as a possible treatment of cSDHs, the topic has gained substantial interest. We sought to summarize the heterogeneity of radiologic measurements, specifically in the evaluation of cSDHs based on literature review.

**Methods:**

In this review, we identified and described the most common radiological methodologies for measurements of cSDH thickness, cSDH volume and of midline shift.

**Conclusion:**

There are numerous published methods on how to evaluate cSDH thickness, cSDH volume and midline shift but no common standard. The definition of measurement methods and reporting standards for MMA embolization in cSDH patients and their validation needs to be addressed.

## Introduction

Chronic subdural hematoma (cSDH) is a frequently occurring pathology in daily neurosurgical practice, with increasing frequency as the population ages [1]. In recent years, embolization of the middle meningeal artery has emerged as a new and promising treatment option for cSDH, either alone or adjuvant to surgical evacuation [2–4]. The aim of this treatment is to devascularize the subdural neomembranes adjacent to the hematoma, which are thought to maintain the subdural blood collection through repeated microhemorrhages secondary to inflammation and neoangiogenesis [3, 5]. Numerous clinical trials evaluating the safety and efficacy of this new treatment method have been recently initiated and some have already been published [4].

A systematic review on 96 studies examining clinical outcomes in patients with cSDH revealed that 39% of the studies examined a radiological outcome measure [6]. These included midline shift, volume and width of the postoperative subdural collection, the latter being the most frequent measure. In addition, clinical indications for surgical treatment of subdural hematomas (SDH) partially rely on radiological assessments [7]; however, there appears to not only be a lack in consensus on which radiological outcome measure to evaluate but also on how to determine it. Various techniques on how to measure SDH thickness, volume, or midline shift can be found in the literature [8–10]. Some of these radiological measures were broadly applied to evaluate extent and space-occupying effect of various intracranial masses but only some studies evaluated their individual application to specifically characterize cSDH [11–13]. This heterogeneity in measurement techniques poses significant barriers to establishing an evidence-based approach to the management of cSDH [3, 6, 14].

In this manuscript, we perform a review of the literature and summarize the heterogeneity of radiological measurements, specifically in the evaluation of cSDH.

## SDH Width and Volume Measurement

A number of different techniques have been published for the measurement of SDH width and volume, which are essential factors for the determination of treatment strategies (conservative vs. surgical) and cSDH monitoring. As manual computer-assisted volumetric analysis is time-consuming and requires a workstation and task-specific software, easy and fast methods for volume estimation were developed.

A simple bedside estimation method of intracerebral hematoma volume, known as the ABC/2 method, was first applied to measure volumes in thalamic hemorrhage [15, 16]. Here, the volume of the hematoma is calculated from maximum width (A), length (B), and height (C) using the formula A × B × C / 2. This method was validated in 118 patients with spontaneous ICH [17]. This group also reported an excellent interrater (intraclass correlation = 0.99) and intrarater (intraclass correlation 0.99) reliability.

The ABC/2 technique was then adapted and validated for use in the measurement of SDH volume [11–13]. A mathematical explanation for the ABC/2 method applied to SDH was first provided in 1977 by Sachs et al. [18] and again in 2005 by Kasner [19]. To our knowledge, only two studies explicitly evaluated this volume measurement technique specifically in patients with cSDH [12, 13].

An overview on methodological details for the measurements of SDH width, length, and depth in the abovementioned studies including patients with acute or cSDH is given in Table 1.

**Table 1:** Overview on methodological details for the measurements of SDH width, length and depth in studies with acute or chronic subdural hematomas

	Reference; year		
	Gebel et al. [11]; 1998	Sucu et al. [12]; 2005	Won et al. [13]; 2018
*Patients*	298 intraparenchymal hematomas and 44 acute subdural hematomas in 244 patients	28 patients with unilateral chronic subdural hematoma	82 patients with 100 chronic subdural hematomas
*Length (A)*	A representative slice at the center of the hematoma Maximum length = linear distance between each corner of the subdural crescent	A1: maximum length on any slice A2: length on slice that is at the center A3: length on slice that has maximum corrected width	Maximum length (anterior to posterior) to each corner of the SDH
*Width (B)*	A representative slice near the center of the hematoma Maximum thickness = from the inner table of the skull perpendicular	B1: maximum width on any slice B2: width on slice at the center B3: corrected width on slice at the center B4: corrected width on slice which has maximum corrected width	Maximum width 90 ° to maximum length in the same slice as length (A)
*Depth (C)*	Number of slices on which hematoma was visible × slice thickness	Number of slices on which hematoma was visible × slice thickness	Number of slices with visible hematoma × thickness of CT-scan or a coronal plane was used
*Volume formula*	A × B × C / 2	A × B × C / 2 with 5 formulas: 1. A1 × B1 × C / 2 2. A2 × B2 × C / 2 3. A2 × B3 × C / 2 4. A3 × B4 × C / 2 5. A1 × B4 × C / 2	A × B × C / 2

Gebel et al. evaluated an adapted ABC/2 technique in 44 acute SDH [11]. Specifically, they suggested to determine the SDH length (A) as the linear distance between each corner of the subdural crescent on a representative slice near the center of the hematoma. The width (B) was then measured as the maximum thickness in cm of hematoma (B) from the inner table of the skull perpendicular to the length. The depth (C) was determined by multiplying the number of slices on which the SDH was visible by the slice thickness listed on the computed tomography (CT) scan. The volume was then calculated using the ABC/2 formula. The volume of the SDH using the adapted ABC/2 method correlated with the computer-assisted method with r = 0.842.

However, Sucu et al. suspected that cSDH differ in shape and size when compared to acute SDH, which makes the validity of the same method in the estimation of chronic hematoma volume questionable [12]. The cSDH are not always symmetrically crescent-shaped. Because of their chronic nature and traction of developing membranes, they may present with asymmetric shapes, such as a comma, pear or lens on axial CT slices. In their study of 22 cSDH patients, Sucu et al. aimed to determine the validity of the ABC/2 technique to measure cSDH volume by comparing it to a computer-assisted volumetric analysis. They created 5 different ABC/2 formulas to evaluate which formula would give the most accurate estimation of hematoma volume compared with the gold standard (Table 1). Even though all 5 formulas showed excellent correlation with the gold standard, the ABC/2 method with measurement of the maximum width and length, which are not necessarily on the same slice, achieved the highest correlation coefficient. Presumably, maximum width was measured perpendicular to length on the slice with maximum width, even though Sucu et al. did not state this explicitly.

In a later study, Won et al. observed a correlation between ABC/2 and computer-assisted volumes with an R^2^ of 0.93 when evaluating 100 cSDH in 82 patients [13]. This is the largest patient group examining volumes specifically in cSDH patients. In contrast to Gebel et al. [11], this group did not use the slice near the center to determine maximum SDH width and length, but used the slice with maximum length to determine maximum width perpendicular to length. In comparison to Gebel et al. and Sucu et al., Won et al. used two techniques for determination of hematoma depth: number of slices with visible hematoma multiplied by thickness of CT scan or a coronal plane; however, they did not report further details on the frequency of each method that was used.

Overall, only 2 studies evaluated the ABC/2 method specifically in patients with cSDH and only the study of Won et al. included a larger number of cases [12, 13]. We could not find any studies that evaluated the performance of the ABC/2 formula and different measurement techniques specifically in postoperative cSDH scans or their accuracy to detect change in cSDH volume or width, even though these parameters are frequently used as primary or secondary outcome measures, as well as for therapy monitoring [4, 6].

However, another technique to determine cSDH volume has emerged over the past years. Neural networks have been used to automatically segment cSDH in a voxel-wise fashion, in order to obtain more accurate volume measurements closer to the gold standard [20, 21]. With broader availability of this technique in the future, it might offer more accurate cSDH volume measurements, especially in longitudinal analyses; however, to our knowledge, only one study has applied this automatic segmentation technique specifically to cSDH patients so far [21]. In their study, Kellogg et al. used a convolutional neural network to segment preoperative and postoperative CT scans achieving an average DICE score of 0.806 on the validation set.

## Midline Shift

**Fig. 1:**
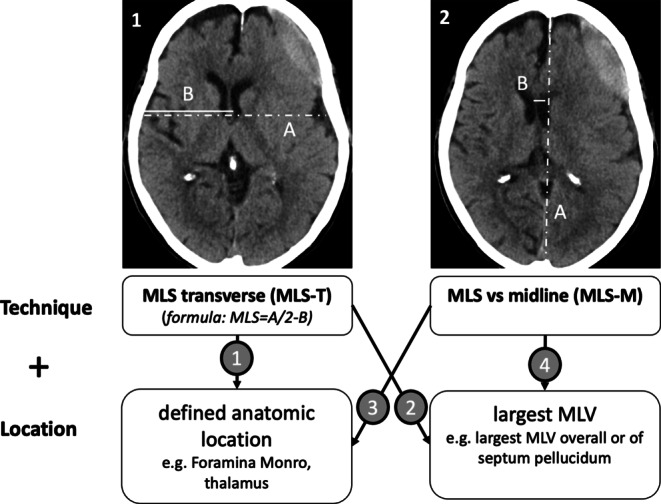
Four major categories of measurement techniques can be defined for the determination of midline shift (*MLS*). (*1*) The (*A*/2-*B*) method, where *A* is the width of the intracranial space and *B* is the distance from the tabula interna to the septum pellucidum at the foramen of Monro, (*2*) the ideal *midline A* is determined as the line between the most anterior and posterior parts of the falx cerebri. *Line B* is drawn perpendicular to *line A* to the septum pellucidum and is then calculated as shift. Each measurement method can be combined with a different predefined measurement location

Another frequent outcome measure in cSDH studies is midline shift (MLS) as a sign of space-occupying effect [6]. Besides cisternal compression and sulcal flattening, MLS is an important indicator of mass effect and can help determine the need for surgical intervention [9]. Different measurement techniques for the estimation of MLS have been published [8, 9, 22], which can be subdivided into four major categories, as shown in Fig. 1. There are two possible measurement techniques, MLS transverse (MLS-T) and MLS vs. midline (MLS-M), that can each be combined either with a specific predefined anatomical measurement location (which indirectly also predefines slice and location of measurement) or the identification of the location with estimated largest MLS (which can be a different slice and location in each patient).

**Fig. 2:**
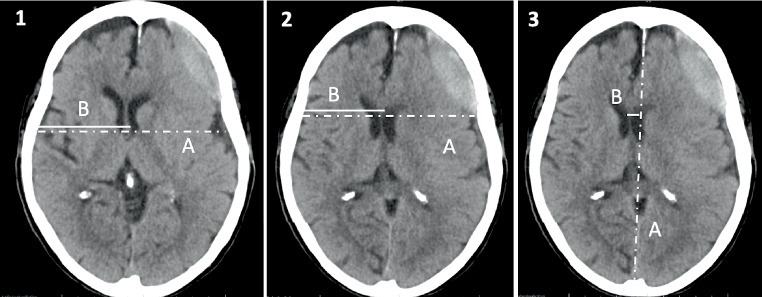
Methods to measure midline shift (*MLS*) in the same patient. (*1*) The (*A*/2-*B*) method, where *A* is the width of the intracranial space and *B* is the distance from the tabula interna to the septum pellucidum at the foramen of Monro (MLS = 0.40 cm). (*2*) The (*A*/2-*B*) method where MLS is measured at the site of largest displacement (MLS = 0.66 cm). (*3*) The ideal *midline A* is determined as the line between the most anterior and posterior part of the falx cerebri. *Line B* is drawn perpendicular to *line A* to the septum pellucidum and is then calculated as shift (MLS = 0.80 cm)

Depending on the applied measurement technique, MLS estimation in cSDH can lead to very different measurements. Fig. 2 displays measurement techniques to determine MLS according to Bullock et al. [9] and two other techniques adapted from Vyvere et al. [8].

As demonstrated by the example in Fig. 2, MLS estimation techniques can lead to very different measurements. Variations, especially when longitudinal studies are analyzed, might also be dependent on slice thickness and patient position or image reconstruction, but also on the etiology, location, and shape of the underlying pathology; however, because the skull is not always symmetric and the patient may not be perfectly aligned during the CT examination, measurement of MLS by first drawing the midline joining the most anterior and posterior visible points on the falx and then measuring the farthest point on the septum pellucidum as perpendicular from the midline might be a more reliable estimate and indeed has shown high interobserver agreement [23]. Moreover, determining the midline is easier than determining the width of the intracranial space, especially when the skull is deformed or removed by surgery or trauma, which is also of high relevance in studies including preoperative and postoperative scans of cSDH patients.

Sucu et al. compared two MLS measurement techniques [22]. They measured MLS at the pineal gland, and the septum pellucidum, both in the preoperative and early postoperative period. The pineal MLS was almost always smaller than the septum pellucidum MLS on both preoperative and postoperative CT images. In addition, MLS exceeding a certain threshold was reported to be associated with restoration of consciousness in patients with cSDH after surgery and also with the occurrence of hemiparesis in patients with unilateral and bilateral cSDH [22, 24]; however, the extent of MLS does not necessarily follow the hematoma width [24]. A possible reason might be that brain atrophy promotes enlargement of extracerebral fluid space. Consequently, the capacity for accumulation of the substantial hemorrhagic collection without critical brain compression and midline shift increases [25].

A recent study published by the Collaborative European NeuroTrauma Effectiveness Research in Traumatic Brain Injury (CENTER-TBI) investigators and participants showed that there are also variations concerning the detection and measurement of MLS when comparing central with local radiological readings [8]; however, to our knowledge, there are no studies systematically comparing estimations from the above listed MLS measurement techniques in cSDH patients and their specific intrarater and interrater variability.

## Special Situations

### Bilateral cSDH

Although bilateral cSDH can be found in approximately 14–25% of cSDH patients, bilateral hematomas are often excluded from cSDH studies, as they pose special challenges concerning the interpretation of radiologic measurements [26, 27]. For example, MLS measurements in patients with bilateral cSDH were reported to be lower than in patients with unilateral cSDH [24, 27]. As the midline is pushed from both sides in patients with bilateral cSDH, MLS cannot be regarded as an adequate marker of space-occupying effect in these patients.

It has also been suggested that bilateral cSDH has a lower incidence of hemiparesis compared to unilateral cSDH, possibly because there is less opportunity for the central brain structures to deviate owing to counterbalance of the mass effect on both hemispheres [27]; however, it was also reported that in patients with bilateral hematoma, the association of hemiparesis and MLS was higher than for unilateral cSDH and the threshold level to cause hemiparesis was lower [24]. In comparison, average volumes and thickness of bilateral cSDH seem to be higher than in unilateral cSDH [26, 28].

### cSDH Close to the Vertex

**Fig. 3:**
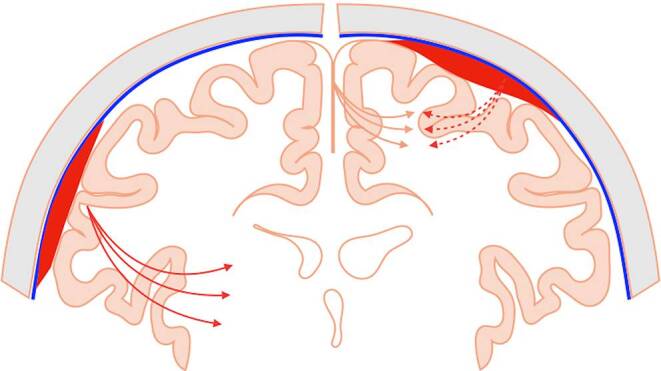
Demonstrates location-specific mass effect of a SDH (coronal slice). When located cranially (*right side*), the affected brain tissue becomes compressed between the SDH and the rigid falx, potentially resulting in more severe symptoms. When located below the falx (*left side*), the brain tissue can shift to the contralateral side, depending on the pliability of the ventricular system and degree of atrophy

Clinically, most patients with cSDH present with headache or mild hemiparesis, even with large MLS; however, symptoms are not only dependent on the size or MLS of the hematoma, but also on its location. The cSDH that are located more cranially are limited by the rigid structure of the falx; in such cases, smaller collections would have a higher risk of leading to more severe symptoms, e.g., hemiparesis of the legs, as the brain is unable to avoid compression (Fig. 3).

**Fig. 4:**
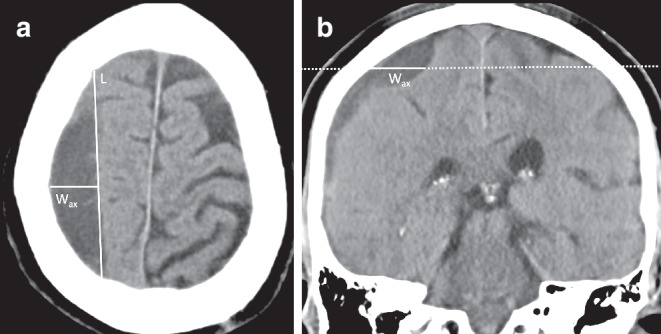
**a** cSDH maximum width measured axial (*W*_*ax*_) perpendicular to maximum length (*L*). **b** Corresponding coronal slice (center of axial slice represented as *dotted line*) with marked W_ax_ measurement (*line*). The width of the SDH measured on a slice close to vertex is greater than it actually is and can overestimate the maximum width of the cSDH

Furthermore, above the superior temporal line, axial CT slices are no longer perpendicular to cranium or cSDH (Fig. 4); they run obliquely because of the curvature of the cranial vault. Therefore, the width of the cSDH on a slice close to vertex is greater than it actually is.

Because the width of hematoma is a variable used in the ABC/2 formula, width measurements conducted close to the vertex of the cSDH might lead to an overestimation of cSDH volume. In their study, Sucu et al. compared different ABC/2 volume estimations including a formula with width measurement that was corrected for the curvature of the cranial vault [12]; however, they did not find an improvement in agreement with corresponding computer-assisted volumes when using the corrected cSDH width. Furthermore, they did not report the absolute difference of hematoma width measurements when comparing different techniques. Also, the effect of these width corrections might be more pronounced in some hematomas than in others and therefore the overall effect might be dependent on the patient population and volumes of the included cSDH. To our knowledge, so far, there are no studies evaluating the differences of cSDH width measurements taken close to the vertex on axial slices and the corresponding measurements on coronal slices and their dependence on cSDH characteristics.

## Conclusion

In summary, radiologic outcome measures and measurement techniques in cSDH are currently very heterogeneous. Validation and direct comparison of different methods specifically for their application in cSDH are scarce. Further studies, especially evaluating the applicability and validity of radiologic outcome measures such as volume, width and MLS in postoperative scans and as treatment monitoring options, as well as their interaction and interrelation are needed.
